# Decomposition and forecasting of colorectal cancer burden attributable to high body mass index and high fasting plasma glucose, 1990–2021: A GBD 2021 study

**DOI:** 10.3389/fnut.2025.1652676

**Published:** 2025-12-17

**Authors:** Ning Gao, Na Yang, Juanjuan Huang

**Affiliations:** 1 Department of Cell Biology and Genetics, Yan’an Medical College of Yan'an University, Yan'an, Shaanxi, China; 2 Department of Obstetrics and Gynecology, Affiliated Hospital of Yan'an University, Yan'an, Shaanxi, China; 3 Department of Reproductive Medicine, Lanzhou University, Second Hospital, Lanzhou, China

**Keywords:** colorectal cancer, decomposition analysis, Global Burden of Disease Study 2021, high body mass index, high fasting plasma glucose

## Abstract

**Background:**

High body mass index (HBMI) and high fasting plasma glucose (HFPG), two key metabolic risk factors, are strongly associated with colorectal cancer (CRC). However, systematic quantification of their impact on the global CRC burden—and trends in related health inequalities—remains limited. Using data from the Global Burden of Disease Study 2021, this study assessed the disability-adjusted life years (DALYs) and deaths attributable to HBMI and HFPG in CRC from 1990 to 2021.

**Methods:**

Decomposition analysis quantified the contributions of population growth, aging, and epidemiological changes. The concentration index and Lorenz curve assessed health inequality trends, and the Estimated Annual Percentage Change (EAPC) measured burden change rates. Burden trends were projected for 2022–2035 using the Bayesian Age-Period-Cohort (BAPC) model. All indicators were stratified by country, Socio-demographic Index (SDI) tier, gender, and age for comparative analysis.

**Results:**

From 1990 to 2021, the burden of CRC attributable to HBMI and HFPG increased, primarily driven by population growth and aging. In high SDI countries, epidemiological changes reduced the burden, while in low- and medium-SDI countries they contributed positively. Although the disease burden remains concentrated in high SDI countries, inequality has declined. Central and Eastern Europe face high DALYs and mortality rates whereas parts of Africa exhibit a lower but rising burden. The burden is primarily concentrated in men over 60. The BAPC model predicts that HBMI- and HFPG-associated DALYs will increase by 47.90 and 41.94%, respectively, while age-standardized DALYs and mortality rates remain relatively stable.

**Conclusion:**

Targeted strategies focusing on metabolic risk management and early CRC screening—especially in low- and middle-SDI countries—are essential to mitigate the projected burden.

## 1 Introduction

Colorectal cancer (CRC) is one of the most prevalent and lethal malignancies worldwide, posing a significant threat to global public health (1–3). According to data from the Global Burden of Disease (GBD) study, in 2020, there were over 1.9 million new cases and more than 900,000 deaths attributed to CRC globally, ranking it third in incidence and second in mortality among all cancer types (4, 5), underscoring its substantial disease burden. In recent years, shifts in lifestyle patterns and the aging population have contributed to a noticeable trend toward earlier-onset of CRC in certain countries and regions. Particularly in high-income countries, the incidence of early-onset CRC (Less than 50 years old) has been steadily rising, prompting heightened concern within the medical community (6).

Although the pathogenesis of CRC has not been fully elucidated, existing research has clearly demonstrated that its development is a complex, multi-stage process involving the combined effects of genetic susceptibility (7), environmental exposures (8), and lifestyle behaviors. Metabolic syndrome is a group of interrelated clinical syndromes, mainly including central obesity, hypertension, elevated fasting blood glucose, and dyslipidemia (9). In multiple epidemiological studies, high body mass index (HBMI) and high fasting blood glucose (HFPG) have been consistently confirmed to be significantly associated with the risk of CRC (10–12). HBMI may promote the occurrence of colorectal cancer by affecting hormone metabolism and the composition of the intestinal microbiota (13–16). Similarly, HFPG, as a pre-diabetic state, can activate key signaling pathways including Smad, WNT, and EGFR, and induce oxidative stress, DNA damage, and dysregulation of apoptosis in chronic hyperglycemia, thus increasing the risk of tumorigenesis (17–20). As the global obesity and diabetes epidemics continue to intensify, the disease burden of CRC posed by these two groups of metabolic risk factors has become a major concern in global public health (21). Existing studies have reported trends in CRC incidence and mortality due to HBMI and HFPG at an aggregate level; however, they provide less insight into the relative contributions of population growth, aging, and epidemiological shifts globally and across countries with different SDI levels (22). In addition, there is no uniform understanding of the differences in the distribution of HBMI and HFPG-associated CRC burden across age, sex, and region, especially for developing countries and low SDI regions, whose place in the global disease transition is often overlooked (23).

Building on data from the GBD 2021, this is the first study systematically integrated multiple research methods, including decomposition analysis, inequality analysis, and forecasting models, to assess the trends and drivers of the CRC burden attributable to HBMI and HFPG globally and across SDI subregions, countries, sexes, and age groups during 1990–2021. The year 2035 coincides with the disease control planning cycle of most countries worldwide and can directly provide practical references for policymakers in setting goals, allocating resources, and conducting prospective impact assessments—effectively serving the forward-looking guidance needs of disease control for this condition. Furthermore, the GBD database integrates long-term time-series data such as population structure, epidemiological dynamics of CRC incidence and mortality, and historical effectiveness of prevention and treatment interventions. Its mature predictive model has been verified to be capable of supporting projections for 10–15 years. Accordingly, this study projects potential trajectories of CRC burden attributable to HBMI and HFPG through 2035, aiming to anticipate future disease trends and support the development of differentiated, stratified metabolic risk mitigation strategies as well as evidence-informed cancer prevention and control policies.

## 2 Materials and methods

### 2.1 Data source

This study is based on the open data platform provided by the Global Burden of Disease Study 2021 (GBD 2021) (http://ghdx.healthdata.org/gbd-results-tool). CRC indicators due to HBMI and HFPG were obtained for the period 1990–2021, including Disability-Adjusted Life Years (DALYs). These include the number of DALYs, deaths, age-standardized DALYs rate (ASDR) and age-standardized mortality rate (ASMR). Corresponding age, sex, country, and Socio-demographic Index (SDI) stratification information was also obtained. The data cover 204 countries and territories worldwide and are organized into five SDI strata and 21 GBD geographic regions. To enhance the readability and clarity of the method, the entire analysis process is presented in a flowchart (Supplementary Figure S1).

### 2.2 Definitions of HBMI and HFPG

This study ultimately included HBMI and HFPG in the analysis. Sufficient existing evidence confirms that both should be incorporated as metabolic risk factors for CRC in the GBD 2021 (24). In the GBD, HFPG is measured as the population’s average fasting glucose—a continuous exposure measure (unit: mmol/L)—defined as any level exceeding the theoretical minimum risk threshold (4.8–5.4 mmol/L). For adults aged ≥20 years, HBMI is defined as BMI > 25 kg/m^2^, based on International Obesity Task Force (IOTF) standards (25).

### 2.3 Decomposition analysis of changes in colorectal cancer burden

To assess the mechanisms driving changes in the burden of CRC over the period 1990–2021, a standard decomposition analysis (DEA) methodology was used to decompose changes in DALYs and deaths into three components: population growth, population aging, and epidemiological changes (which represent the change in age-specific risk rates due to factors such as shifts in risk factor prevalence, advances in medical care, and changes in screening and diagnosis). The decomposition (1990 as the base) was performed using the method by Das Gupta, which partitions the difference in rates between two time points into the contributions of specified factors, accounting for their non-additive interactions (26). This method summarizes the contribution of various factors to the observed changes by algebraically isolating the standard impact of each contributing multiplicative factor. The analysis was conducted at the global level and in each of the five SDI subregions, and the results are presented in stacked bar charts (27).

### 2.4 Health inequalities assessment

In this study, the Concentration Index (CI) and Lorenz Curve were used to measure the equity of the distribution of CRC DALYs and mortality due to HBMI and HFPG across countries with different SDI levels. The 204 countries were ranked based on the population-weighted SDI rankings, and the CI values and curve patterns were plotted for 1990 and 2021, respectively. The slopes of DALYs rates or mortality rates on the SDI series were further assessed by weighted least squares regression (Robust Linear Model, RLM) to characterize the expansion of burden differentials with the level of development (28).

### 2.5 Burden assessment and trend analysis at the national level

At the country level, data on CRC age-standardized DALYs rates and mortality rates due to HBMI with HFPG in 2021 were extracted, sorted by country, and a heat map of the global distribution was produced per 100,000 population, reflecting spatial heterogeneity. To characterize temporal trends in CRC DALYs rates and mortality from 1990 to 2021, EAPC was calculated using linear regression on natural log-transformed ASDR or ASMR values. The trend is defined as significantly increasing if the 95% uncertainty interval (95% UI) of the EAPC is entirely positive; conversely, it is defined as significantly decreasing. All countries were included in the analysis, and the results were visualized by color charts.

### 2.6 Burden assessment and trend analysis at the national level

To predict the global burden of CRC attributable to HBMI and HFPG, and to provide a basis for precise prevention and control, this study employed the Bayesian Age-Period-Cohort (BAPC) model framework. The model was implemented using the “BAPC” R package, with data from the GBD 2021 and population data from the Institute for Health Metrics and Evaluation (IHME) (29). The BAPC model addresses the inherent identifiability problem of APC models by applying smoothing priors to the age, period, and cohort effects, providing a unique and stable solution based on the principle of parsimony.

This study used data from 1990 to 2021. The timeline was divided into consecutive period groups (j) at 5-year intervals, and the study population was also divided into age groups (i) at corresponding 5-year intervals. Five-year intervals are commonly employed in BAPC model applications for chronic diseases, as they strike a balance between model stability and analytical resolution while maintaining alignment with the characteristics of GBD data. Birth cohorts (k) were determined based on the relationship between period and age (k ≈ central year of the period minus the central year of the age group). The model assumes that the number of events follows a Poisson distribution, with its core structure defined as follows:


$$\log\;(E_{\mathrm{ij}})=\log(N_{\mathrm{ij}})+\mu +\alpha_{i}+\pi_{j}+\gamma_{k}$$

In the formula, *E_ij_* represents the expected number of events in age group i and period group j; *N_ij_* denotes the corresponding exposed person-years, and the logarithm of *N_ij_* is included as an offset term; *μ* is the intercept; *α_i_*, *π_j_*, and *γ_k_* (determined by *i* and *j*) are the main effects of age, period, and cohort, respectively (relative to their respective reference groups).

Built on the Bayesian generalized linear model, the BAPC model treats age, period, and cohort effects as continuous changing processes. It enhances the robustness of estimates and the accuracy of predictions through smoothing via second-order random walks. The model adopts the Integrated Nested Laplace Approximation (INLA) method to efficiently calculate marginal posterior distributions, avoiding the mixing and convergence issues associated with Markov Chain Monte Carlo techniques (30). Suitable for analyzing complex temporal trends and population structure data, this model has been widely applied in epidemiological burden studies (31).

#### 2.6.1 Model specification and validation

In this study, the smooth parameters of the random walk model were set using the Penalized Complexity prior (PC prior), which ensures model flexibility while controlling complexity. To evaluate the predictive performance of the model, we fitted the model using data from 1990 to 2010 and extrapolated the predictions to 2021. Subsequently, the predicted values were compared with the actual observed data for analysis. By integrating the age-standardized rates (ASR) predicted by the model with the actual ASR, four indicators—mean squared error (MSE), mean absolute error (MAE), mean absolute percentage error (MAPE), and coefficient of determination (R^2^)—were used to comprehensively assess the predictive accuracy of the model. The final validation results were presented in groups according to indicator type, gender, and other dimensions. To achieve smooth estimation and solve the identification problems existing in the traditional age-period-cohort (APC) model, the age effect *α_i_*, period effect *π_j_*, and cohort effect *γ_k_* were all assigned a second-order random walk (RW2) prior distribution. This prior assumes that the second-order differences of each effect follow a normal distribution with a mean of zero, effectively capturing the smooth nonlinear trends in the data. For the precision parameters and intercept term *μ* in the RW2 model, the standard weak information prior recommended by the BAPC software package was used. The posterior distribution estimation and statistical inference of the model parameters were completed in the R language environment by calling the BAPC package, and the calculation process was based on the Integrated Nested Laplace Approximation (INLA) method, significantly improving computational efficiency. The 95% uncertainty intervals (95% UI) is derived directly from the posterior sampling results of the model, reflecting the uncertainty of parameter estimates caused by data variability and model structure; for decomposition analysis and CI calculation, the 95% UI is obtained through 1,000 bootstrap resampling iterations, which accounts for the random error in the decomposition of burden drivers and the calculation of equity indicators.

### 2.7 Statistical analysis

All data processing, statistical calculations, and graphical visualization in this study were performed using R software (version 4.4.1), and some trend analyses were conducted using the Joinpoint regression program. All statistical tests were two-sided and the significance level was set at *p* < 0.05.

## 3 Results

### 3.1 Decomposition analysis of changes in colorectal cancer DALYs and deaths due to HBMI and HFPG

#### 3.1.1 Global trends

**Figure 1:**
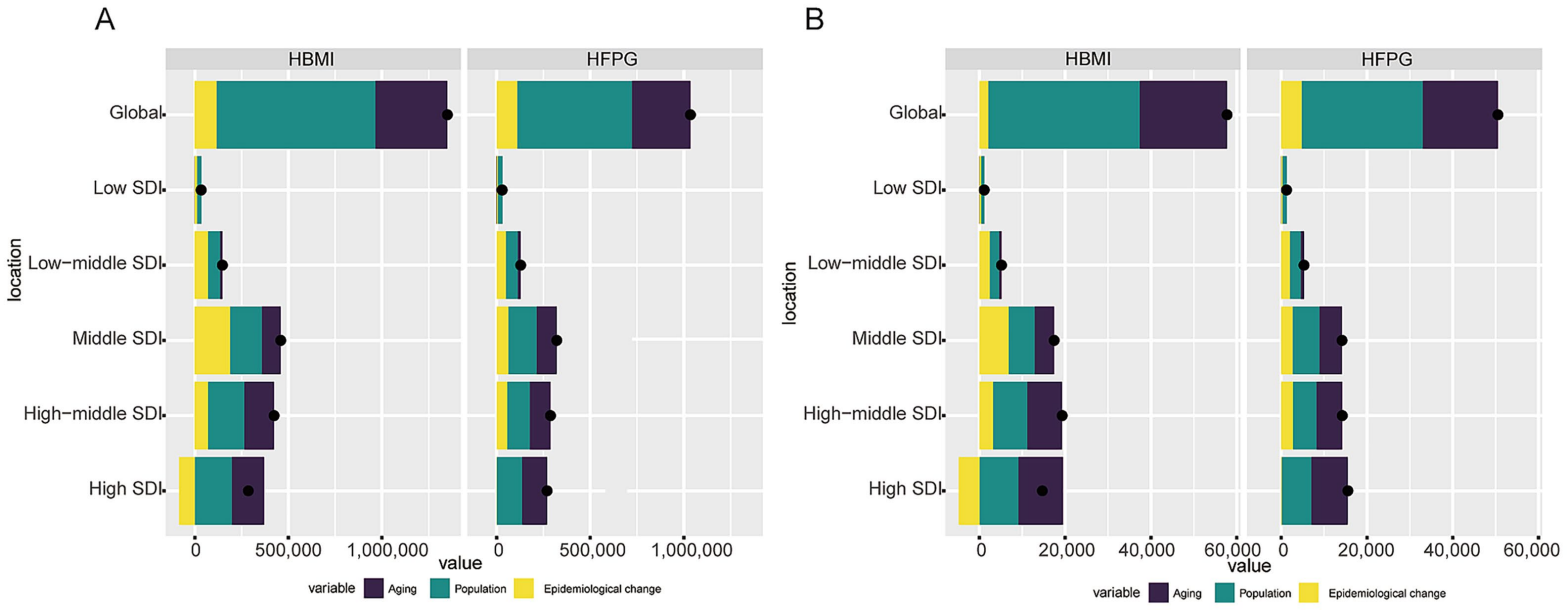
Analysis of DALY changes and deaths from CRC caused by HBMI and HFPG. **(A)** Decomposition of the increase in DALYs due to HBMI versus HFPG for CRC between 1990 and 2021, shown globally versus by SDI region. Contributions are categorized into population growth, population aging, and epidemiological changes. **(B)** Decomposition of the increase in CRC deaths due to HBMI versus HFPG between 1990 and 2021, shown by global versus SDI region.

Between 1990 and 2021, the total global DALYs for CRC caused by HBMI increased by 1349622.04. Of these, population growth was the main driving factor, accounting for a 62.92% increase in DALYs, followed by population aging, which contributed a 28.52% increase, and epidemiological changes, which accounted for an additional 8.56%. Similarly, the rise in DALYs due to HFPG amounted to 1035207.56, with population growth and aging remaining the dominant factors (increasing DALYs by 59.32 and 30.16%, respectively), while epidemiological changes contributed 10.52% of the increase (Figure 1A; Supplementary Table S1). In terms of deaths, the increase in CRC deaths due to HBMI was 57732.24; of this increase, 61.10% was attributed to population growth, 35.32% to aging, and only 3.58% to epidemiological changes. In contrast, the increase in deaths associated with HFPG totaled 50514.40, again primarily driven by population growth (55.89%) and aging (34.66%), with epidemiological changes contributing 9.45% (Figure 1B; Supplementary Table S1).

#### 3.1.2 Regional disparities

Between 1990 and 2021, the drivers of changes in DALYs and deaths differed markedly across SDI subregions. In high SDI regions, due to epidemiological changes, DALYs and deaths caused by HBMI showed negative growth, with DALYs decreasing by 29.43% and deaths by 32.8%, suggesting that the district may be making progress in risk control or early screening. Meanwhile, aging remains the primary factor in the increase of DALYs in the district, with HBMI increasing by 60.32% and HFPG by 50.10%. In high-middle SDI regions, the contributions of the three factors were relatively balanced, with the role of epidemiological changes being particularly significant. HBMI-related DALYs increased by 16.56% and mortality by 16.36%, while HFPG-related DALYs increased by 19.48% and deaths by 19.07%. In middle and low-middle-SDI areas, the growth in DALYs was primarily driven by a combination of population growth and epidemiological changes. For example, in lower-middle SDI regions, 44.31% of the increase in DALYs due to HBMI was attributed to population growth, while 47.35% was due to epidemiological changes. In low-SDI areas, while population growth remains dominant, such as a 68.98% increase in DALYs due to HBMI and 86.81% increase due to HFPG. However, the impact of epidemiological changes could not be ignored, with DALYs increasing by 19.02% due to HFPG and 35.06% due to HBMI. In low-SDI regions, the aging factor reduced DALYs due to HBMI and HFPG by 4.05 and 5.83%, and their mortality contribution by 4.1 and 4.15%, respectively. Although the GBD framework estimates independent effects, HBMI and HFPG are often comorbid. Therefore, the presented burdens should be interpreted as the potential burden attributable to each risk factor individually, with the understanding that there may be residual overlap due to shared pathophysiological pathways (Figure 1; Supplementary Table S1).

### 3.2 Health inequalities analysis: trends in the SDI distribution of colorectal cancer DALYs and deaths due to HBMI and HFPG between 1990 and 2021

**Figure 2:**
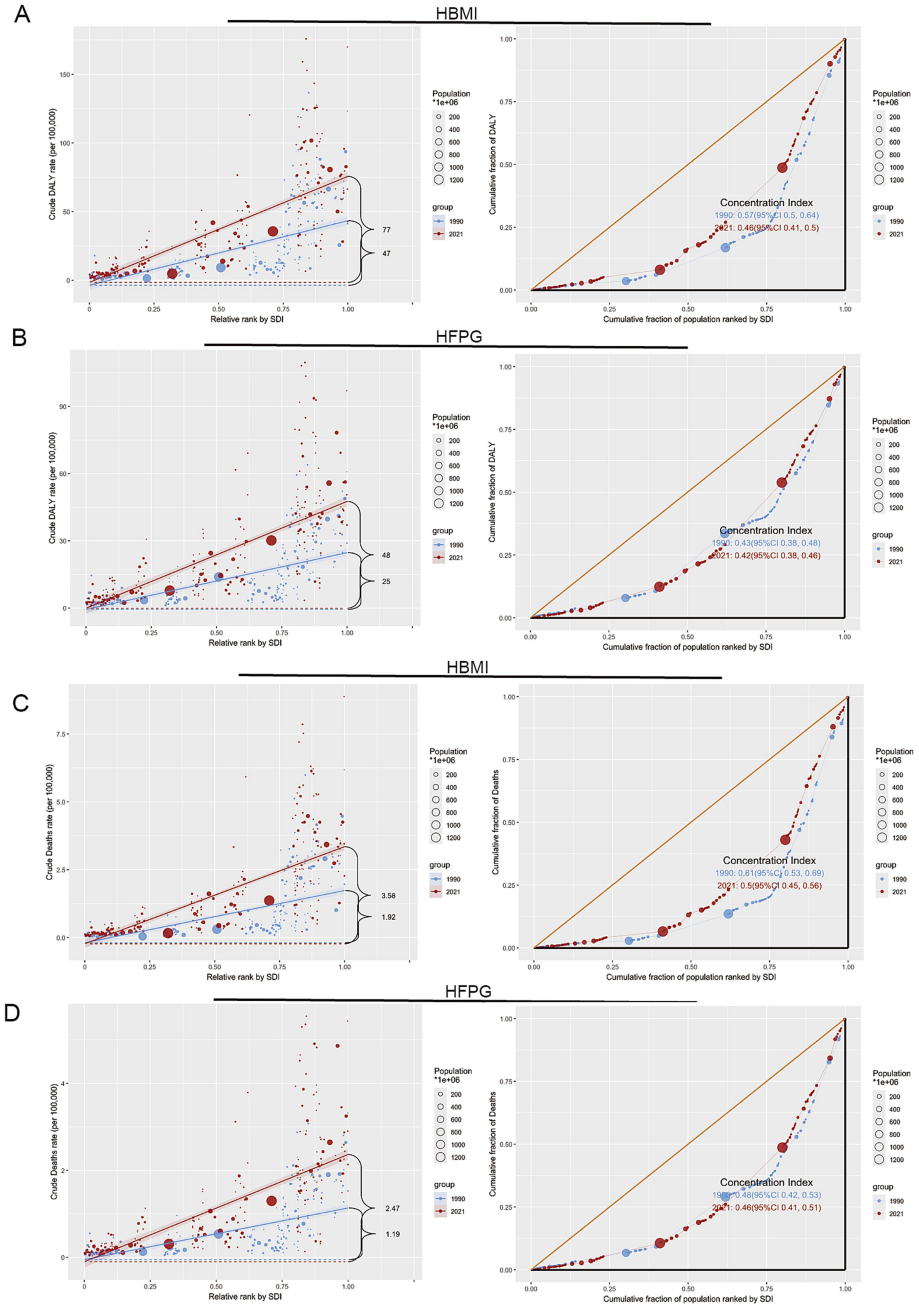
Health inequalities analysis. **(A)** SDI distribution of DALYs for HBMI-associated CRC. **(B)** SDI distribution of DALYs for HFPG-associated CRC. **(C)** SDI distribution of HBMI-associated CRC DALYs. **(D)** SDI distribution of CRC mortality associated with HFPG. The left panel shows the SII analysis, the right panel shows the concentration index analysis.

Health inequality analysis shows that the slope index of inequality (SII) shifted from 47.0156 (95% CI: 39.5320–54.4992) in 1990 to 77.1772 (95% CI: 69.7443–84.6101) in 2021, while the concentration index decreased from 0.5670 (95% UI: 0.4972–0.6368) in 1990 to 0.4575 (95% UI: 0.4112–0.5038) in 2021. A positive concentration index indicates that the burden is disproportionately concentrated among populations with higher socio-demographic status. In 2021, the Lorenz curve was closer to the equality line, with a significantly reduced deviation compared to 1990, further reflecting the expansion of the DALY burden in the SDI dimension (Figure 2A; Supplementary Table S2). Figure 2B shows that, in 1990 and 2019, the SII of DALYs for CRC due to HFPG were 25.3361 (95% CI: 21.6931–28.9791) and 47.6766 (95% CI: 42.8305–52.5226), respectively. The concentration index was 0.4309 (95% UI: 0.3836–0.4782) in 1990 and 0.4188 (95% UI: 0.3810–0.4567) in 2021, with a limited decrease. The 2021 Lorenz curve is close to the 1990 pattern, with a larger deviation from the equality line (Figure 2B; Supplementary Table S2).

The SII of deaths for CRC due to HBMI was 1.9225 (95% CI: 1.6081–2.2369) in 1990 and increased to 3.5760 (95% CI: 3.2231–3.9290) in 2021. The concentration index was 0.6084 (95% UI: 0.5294–0.6873) in 1990, declining to 0.5046 (95% UI: 0.4500–0.5593) by 2021. Figure 2C illustrates the high concentration of mortality in the high SDI countries in 1990 and the significant increase in mortality in the middle SDI countries through 2021. For the incidence of HFPG-related mortality, SII shift from 1.1878 (95% CI: 0.9995–1.3761) in 1990 to 2.4659 (95% CI: 2.2132–2.7187) in 2021, the concentration index was 0.4785 (95% UI: 0.4237–0.5333) in 1990 and 0.4627 (95% UI: 0.4145–0.5109) in 2021, with little change. Figure 2D shows that mortality is concentrated in high SDI countries, with smaller increases in low SDI countries (Figure 2D; Supplementary Table S2).

### 3.3 Global burden description

#### 3.3.1 Analysis of differences in the burden of DALYs at the country level

**Figure 3:**
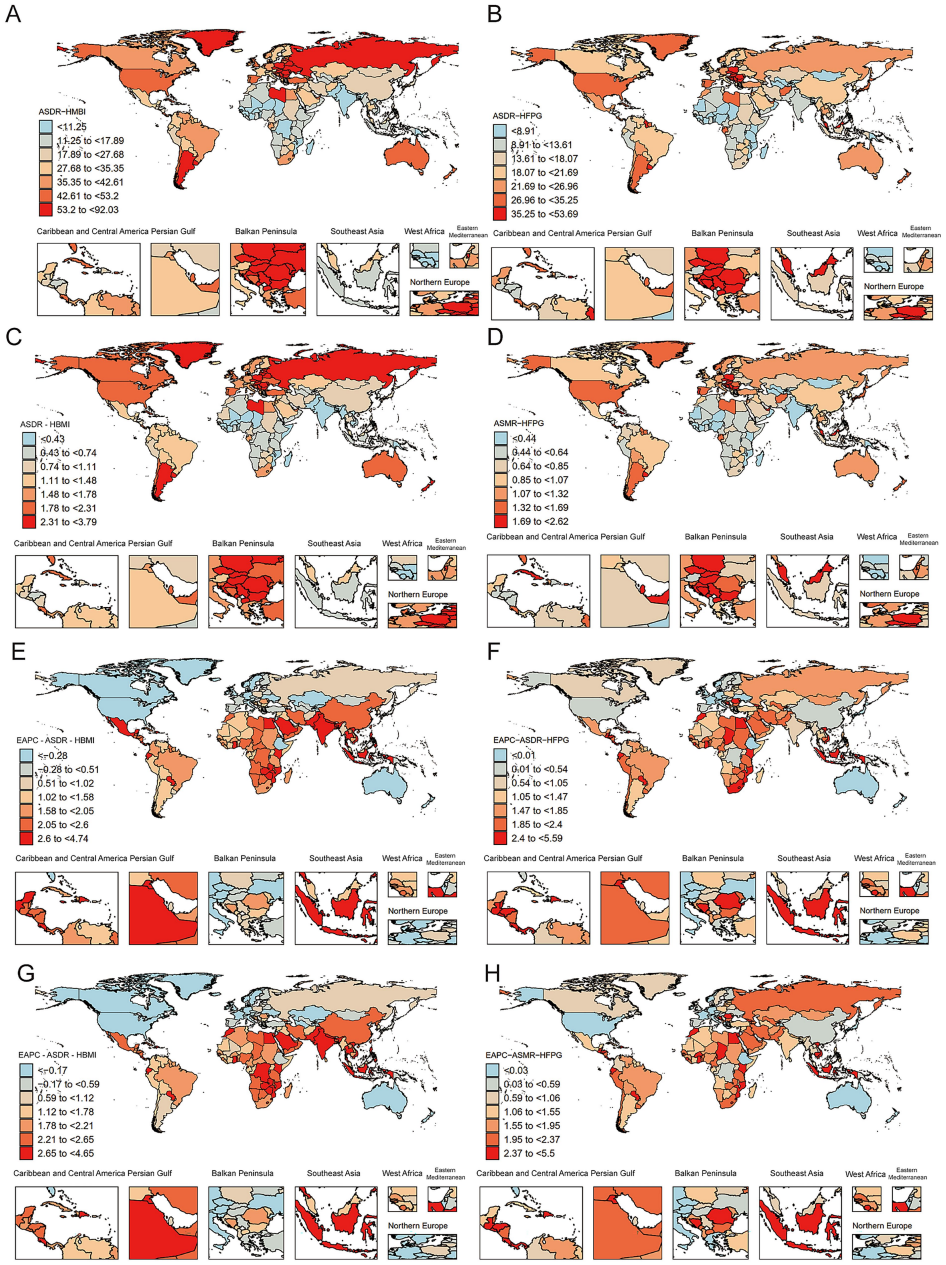
Analysis of differences in the burden of DALYs, deaths, and EAPC trends at the country level. **(A)** Distribution of CRC DALYs rates due to HBMI in 2021 by country (in units per 100,000 population). Darker colors indicate higher burden. **(B)** Distribution of CRC DALYs rates due to HFPG by country in 2021 (per 100,000 population). Darker colors indicate higher burden. **(C)** Distribution of CRC mortality due to HBMI by country in 2021 (in units per 100,000 population). Darker colors indicate a higher burden of CRC deaths due to HBMI. **(D)** Distribution of CRC mortality due to HFPG by country in 2021 (per 100,000 population). **(E)** EAPC distribution of CRC DALY rates due to HBMI by country, 1990–2021. Darker colors indicate a faster rate of increase in the burden of DALYs (higher EAPC values), blue represents a downward trend, and red indicates an increasing trend, and the figure illustrates differences in the distribution of countries with significant increases or decreases. **(F)** EAPC distribution of CRC DALYs rates due to HFPG by country, 1990–2021. **(G)** EAPC distribution of CRC mortality rates due to HBMI by country, 1990–2021. **(H)** EAPC distribution of CRC mortality rates due to HFPG by country, 1990–2021.

Figures 3A,B illustrate the differences in the rates of CRC DALYs due to HBMI with HFPG by country in 2021. Overall, the burden of DALYs was significantly higher in high SDI countries, such as Europe and the United States, than in low and medium SDI countries, but individual developing countries also exhibited relatively high burdens, indicating a trend toward global spread of risk factors. Regarding DALYs associated with HBMI, the countries with the highest DALY rates included Hungary 92.03/100,000 (95% UI:41.88–149.98), and Slovakia 82.15/100,000 (95% UI:37.40–131.78), which generally have high rates of obesity. In contrast, South Asian countries such as India 5.35/100,000 (95% UI:2.10–8.38) and Bangladesh 3.41/100,000 (95% UI:1.21–6.04) had low rates of DALYs, which may be related to their relatively low average BMI levels (Figure 3A; Supplementary Table S3).

The top countries in terms of HFPG-related DALYs include Hungary 53.69/100,000 (95% UI:26.01–85.07), Barbados 52.17/100,000 (95% UI:26.75–84.71), and Bulgaria 50.61/100,000 (95% UI:24.64–81.81). In contrast, some sub-Saharan African countries, such as Malawi 3.88/100,000 (95% UI:1.81–6.42), Mozambique 3.84/100,000 (95% UI:1.71–6.55), and Chad 8.91/100,000 (95% UI:4.27–14.40), are less burdened. Notably, the rate of DALYs in China was 24.22/100,000 (95% UI: 9.98–40.69) for the burden associated with HBMI and 20.25/100,000 (95% UI: 9.93–31.64) for HFPG, both of which were in the middle of the range. The DALY rates in Taiwan, China were 44.71/100,000 (95% UI:18.68–71.98) (HBMI) and 38.60/100,000 (95% UI:19.18–59.03) (HFPG), respectively, which were higher than those in mainland China, suggesting that there are regional differences in health risk control (Figure 3B; Supplementary Table S3).

#### 3.3.2 Analysis of differences in the burden of deaths at country level

Central European and Latin American countries such as Hungary 3.79/100,000 (95% UI:1.72–6.19) and Slovakia 3.53/100,000 (95% UI:1.61–5.66) ranked among the top countries in terms of CRC ASMR attributable to HBMI. This indicates that the association between HBMI and CRC deaths was particularly significant in these countries. In addition, small countries such as Monaco 3.19/100,000 (95% UI:1.41–5.43) and Barbados 3.05/100,000 (95% UI:1.32–5.11) also exhibit high mortality rates, suggesting that they may be influenced by lifestyle, westernized diets or rising body mass index. In contrast, several sub-Saharan African countries have significantly lower associated mortality burdens. For example, countries such as Malawi 0.24/100,000 (95% UI:0.09–0.41), Mozambique 0.18/100,000 (95% UI:0.06–0.32) and Chad 0.29/100,000 (95% UI:0.12–0.49) have CRC mortality rates below 0.30/100,000 due to HBMI (Figure 3C; Supplementary Table S3).

In terms of HFPG-related deaths, countries with high mortality rates included Barbados 2.62/100,000 (95% UI:1.35–4.21), Hungary 2.47/100,000 (95% UI:1.19–3.94), Poland 2.45/100,000 (95% UI:1.24–3.68), and Croatia 2.36/100,000 (95% UI:1.16–3.69), all with more than 2.0/100,000. In contrast, the countries with the lowest HFPG-related mortality rates were concentrated in sub-Saharan Africa, including Malawi 0.19/100,000 (95% UI:0.09–0.31), Gambia 0.21/100,000 (95% UI:0.10–0.35) and Mozambique 0.21/100,000 (95% UI:0.09–0.36), among others (Figure 3D; Supplementary Table S3).

#### 3.3.3 Country-level EAPC trend analysis: rate of change in DALYs and mortality, 1990–2021

Figures 3E–H shows the EAPC in the rate of CRC DALYs due to HBMI with HFPG versus mortality from 1990 to 2021, revealing trends in the burden by country. The fastest growing countries in terms of HBMI-related DALYs included Vietnam, Lesotho, and Zimbabwe. Relatively, some high-income countries showed a decreasing trend in the rates of DALYs associated with HBMI. For example, Austria (EAPC = −1.88, 95% UI: −1.94 to −1.82), the Czech Republic (−1.81, 95% UI: −1.99 to −1.63), and Germany (−1.65, 95% UI: −1.77 to −1.54) (Figure 3E; Supplementary Table S3). In terms of HFPG-related DALYs, Lesotho, Egypt and Georgia ranked high. The country with the most significant decline in HFPG-related DALYs was Singapore (−2.12, 95% UI: −2.33 to −1.91) (Figure 3F; Supplementary Table S3). In terms of mortality, countries with fast growing EAPC values for HBMI-associated CRC deaths included Viet Nam, Lesotho & Cabo Verde (Figure 3G; Supplementary Table S3). The rapid increase in HFPG-related deaths was also concentrated in low- and middle-income countries such as Egypt, Lesotho, and Georgia (Figure 3H; Supplementary Table S3). In contrast, high-income countries such as Singapore, Switzerland, Japan, and Israel showed significant declines in mortality with negative EAPCs.

### 3.4 Burden analysis of DALYs and deaths by age stratification

#### 3.4.1 Burden analysis of DALYs by age stratification

**Figure 4:**
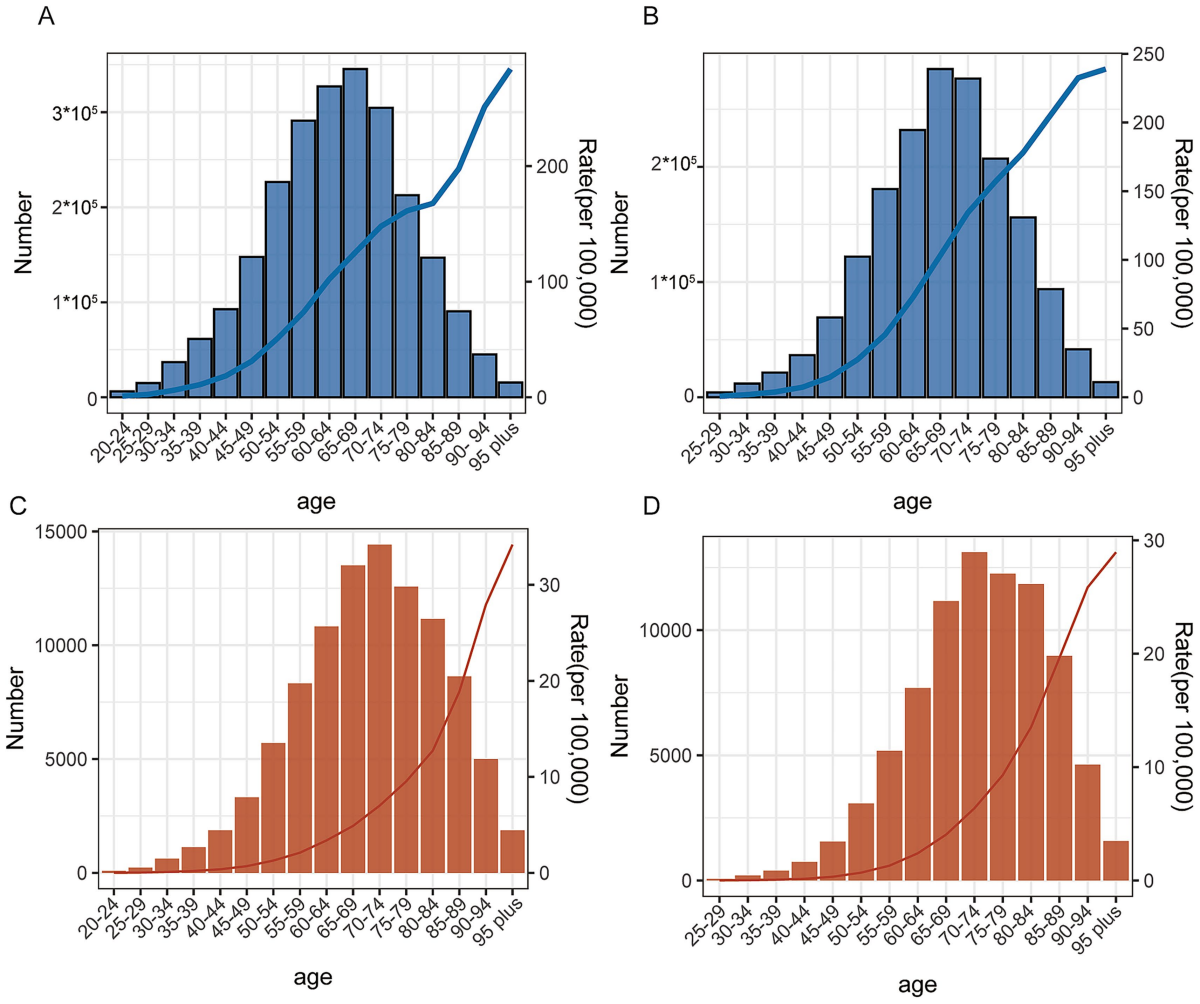
Burden analysis of DALYs and death by age stratification. **(A)** Distribution of the number of CRC DALYs versus the rate of DALYs due to HBMI across age groups. **(B)** Distribution of the number of CRC DALYs versus the rate of DALYs due to HFPG across age groups. **(C)** Distribution of CRC deaths versus mortality due to HBMI by age. **(D)** Distribution of CRC deaths versus mortality due to HFPG by age.

Figures 4A,B show the trends in the distribution of the number of CRC DALYs (left) and the rate of DALYs (right, in units per 100,000 population) due to HBMI and HFPG by age in 2021, respectively. In terms of HBMI-associated DALYs, the number of DALYs continued to increase with age, from 6,149 person-years (95% UI: 2,465-9,661) in the 20–24 year old group to a peak of 345,429 person-years in the 65–69 year old group (95% UI: 151,213-544,676). It then declined slightly but remained at 15,473 person-years (95% UI: 6,184-25,564) in the 95 years and older group. The corresponding rate of DALYs increased gradually from 1.03/100,000 (95% UI: 0.41–1.62) at ages 20–24 to 283.90/100,000 (95% UI: 113.4723–469.05) at age 95 years or older (Figure 4A; Supplementary Table S4).

Trends were similar for HFPG-associated DALYs. The number of DALYs in 25–29 year olds was 4,178 person-years (95% UI: 2,020-6,594), peaked at 284,822 person-years (95% UI: 143,759–431,537) in 65–69 year olds, and remained at 13,019 person-years (95% UI: 6,231––20,027) at age 95 years and older. The rate of DALYs also gradually increased from 0.71/100,000 (95% UI: 0.34–1.12) in the 25–29 years old group to 238.88/100,000 (95% UI: 114.34–367.44) in the 95 years old and above group (Figure 4B; Supplementary Table S4).

#### 3.4.2 Analysis of colorectal cancer mortality burden by age stratification

Figures 4C,D show the distribution trend of the number of CRC deaths and mortality due to HBMI and HFPG in different age groups in 2021, respectively. Regarding the number of deaths associated with HBMI, the number of deaths continued to increase with age, from 89 cases (95% UI: 36–140) in the 20–24 year old group to 14,418 (95% UI: 6,254-22,960) in the 70–74 year old group. Thereafter, there was a slight decline, but there were still 1,863 cases (95% UI: 741–3,088) in the 95 years and older group. As for the mortality rate, it was 0.01/100,000 (95% UI: 0.006–0.023) for 20–24 year olds and peaked at 34.19/100,000 (95% UI: 13.60–56.67) in older age (Figure 4C; Supplementary Table S4). This suggests that the number and risk of HBMI-associated CRC deaths doubly accumulate in the middle and old age stages.

Trends were similar in the number of HFPG-related deaths. The number of deaths among 25–29 year olds was approximately 65 (95%UI: 31–102), peaking at 13,108 (95% UI: 6,554–20,243) at 70–74 years of age, and remaining at 1,577 (95% UI: 754–2,422) over 95 years of age. The corresponding mortality rate increased progressively from 0.01/100,000 (95% UI: 0.005–0.0110) in the 25–29 year olds, increased to 6.38/100,000 (95% UI: 3.18–9.83) in the 70–74 age group. The high burden was maintained in the advanced age group although it decreased slightly (Figure 4D; Supplementary Table S4). This suggests that the impact of HFPG on the risk of CRC mortality is particularly prominent in the 60+ age group.

### 3.5 Gender-stratified colorectal cancer burden analysis

**Figure 5:**
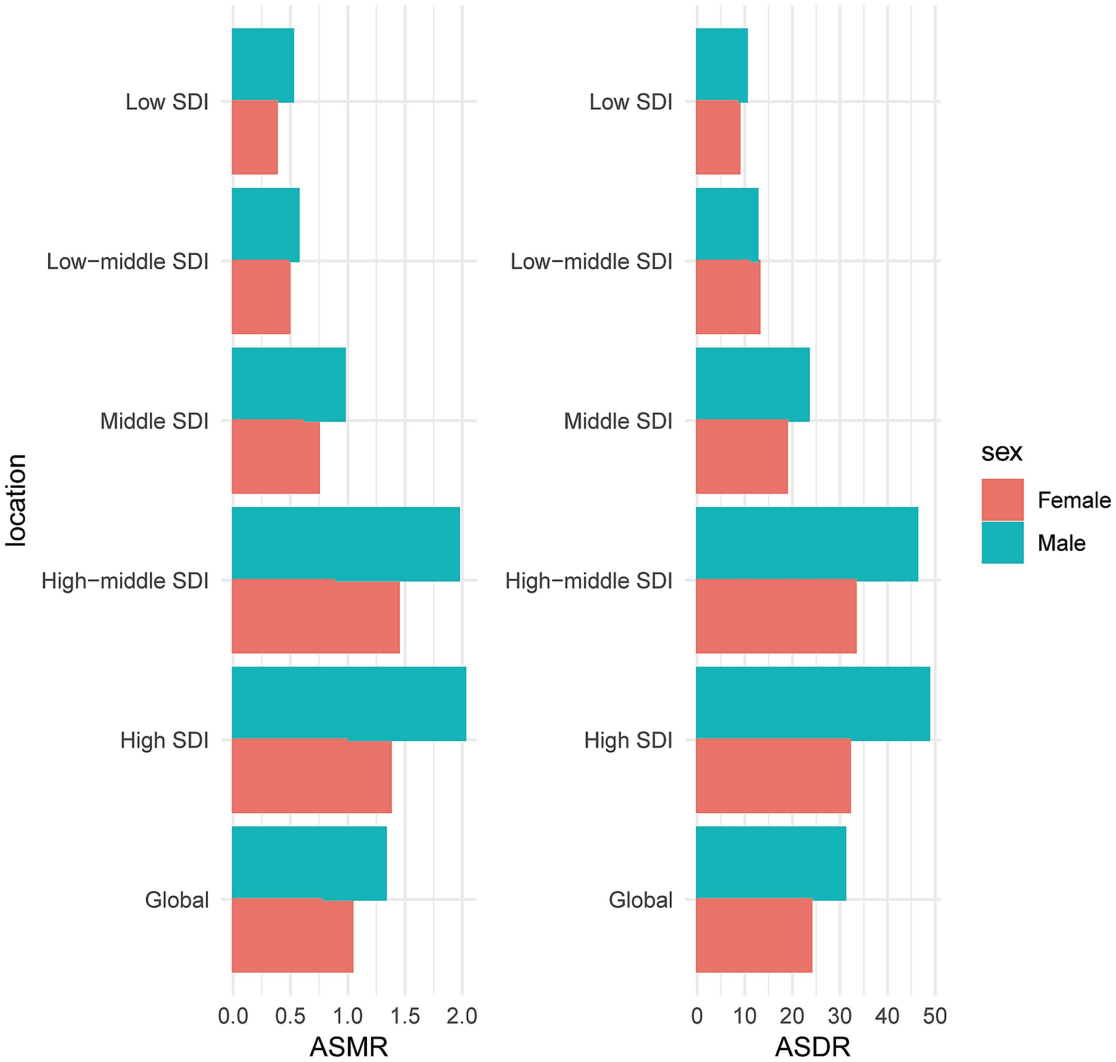
Gender-stratified CRC burden analysis. Distribution of ASMR and ASDR for CRC due to HBMI with HFPG across gender and SDI stratified countries.

Figure 5 shows the distribution of age-standardized DALYs rates and age-standardized mortality rates for CRC due to HBMI vs. HFPG across sexes, respectively. The data showed that ASDR and ASMR were significantly higher in men than in women, both globally and in each SDI stratification region. In terms of ASDR, globally, the ASDR for CRC due to HBMI was 31.1 per 100,000 for men and 23.96 per 100,000 for women, and the ASDR due to HFPG was 25.76 per 100,000 for men and 15.59 per 100,000 for women. Similar differences were observed under each SDI stratum, especially in high SDI and high-middle SDI countries, where the gap between men and women was more pronounced (Figure 5; Supplementary Table S5). Overall, men accounted for a higher proportion of CRC DALYs and mortality burden associated with HBMI with HFPG.

In terms of mortality (ASMR), the global mortality rate due to HBMI was 1.33/100,000 (95% UI: 0.57–2.13) for males, which was significantly higher than that for females at 1.04/100,000 (95% UI: 0.45–1.65). HFPG-related mortality also showed gender differences, with 1.25/100,000 (95% UI: 0.64–1.90) in men and 0.77/100,000 (95% UI: 0.39–1.16) in women. Particularly in the high SDI countries, the mortality rate induced by HBMI amounted to 2.02/100,000 (95% UI: 0.87–3.19) in men, which was much higher than that of 1.37/100,000 (95% UI: 0.60–2.19) in women (Figure 5; Supplementary Table S5).

### 3.6 Subgroup description: analysis of colorectal cancer burden globally, in SDI strata and 21 GBD regions

**Figure 6:**
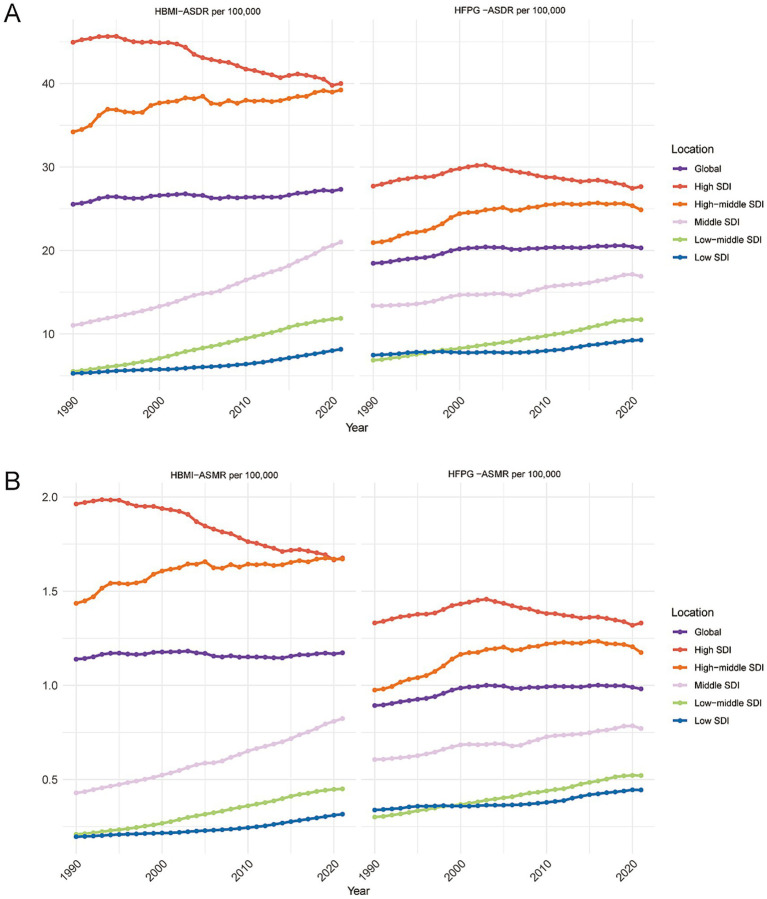
Subgroup description. **(A)** Distribution of ASDR and ASMR for CRC due to HBMI in countries stratified by SDI. **(B)** Distribution of ASDR and ASMR for CRC due to HFPG, stratified by SDI.

Based on the results in Figure 6 and Tables 1, 2, we analyzed the differences in ASDR, ASMR, and EAPC for CRC attributable to HBMI with HFPG between 1990 and 2021. Globally in 2021, the HBMI related ASMR was 1.17/100,000 (95% UI:0.51–1.87) and the ASDR was 27.33/100,000 (95% UI:11.80–43.37). The corresponding EAPC values were 0 (95% CI: −0.04 to 0.04) for ASMR and 0.12 (95% CI: 0.08 to 0.16) for ASDR, indicating a slow but increasing global burden. At the SDI level, high-SDI countries exhibited significantly higher ASMR: 1.68/100,000 (95% UI:0.73–2.66) and ASDR: 40/100,000 (95% UI:17.48–62.93) compared to low-SDI countries. However, the EAPC trends diverged markedly: high-SDI countries showed a significant decrease in both ASMR (EAPC: −0.64; 95% CI: −0.69 to 0.59) and ASDR (EAPC: −0.48; 95% CI: −0.52 to 0.43), whereas low-SDI countries experienced a substantial increase, with EAPC values of 1.5 (95% CI: 1.38–1.62) for ASMR and 1.33 (95% CI: 1.22–1.45) for ASDR. Among the 21 GBD regions, Central Europe had the highest ASMR (3.03/100,000; 95% UI:1.35–4.85) and ASDR (68.94/100,000; 95% UI:30.82–110.6), while South Asia had the lowest (ASMR: 0.20/100,000; 95% UI:0.08–0.32; ASDR: 5.67/100,000; 95% UI:2.22–8.92). The most rapid increase in EAPC was observed in Southeast Asia, with value of 3.04 (95% CI:2.93–3.15) for ASMR and 2.84 (95% CI:2.72–2.97) for ASDR, highlighting a sharp rise in BMI-related burden in that region (Figure 6A; Table 1).

**Table 1:** ASMR and ASDR attributable to HBMI in 2021, and their trends from 1990 to 2021 by SDI regions and global burden of disease locations.

Location	ASMR in 2021(per 100,000)	EAPC of ASMR, 1990 to 2021 (95%CI)	ASDR in 2021 (per 100,000)	EAPC of ASDR, 1990 to 2021 (95%CI)
Global	1.17 (0.51 to 1.87)	0 (−0.04 to 0.04)	27.33 (11.8 to 43.37)	0.12 (0.08 to 0.16)
High SDI	1.68 (0.73 to 2.66)	-0.64 (−0.69 to −0.59)	40 (17.48 to 62.93)	-0.48 (−0.52 to −0.43)
High-middle SDI	1.67 (0.72 to 2.66)	0.4 (0.32 to 0.48)	39.23 (16.94 to 62.34)	0.31 (0.24 to 0.37)
Middle SDI	0.82 (0.35 to 1.32)	2.13 (2.11 to 2.16)	21.01 (8.93 to 33.45)	2.1 (2.07 to 2.14)
Low-middle SDI	0.45 (0.19 to 0.71)	2.72 (2.65 to 2.79)	11.87 (5 to 18.73)	2.69 (2.63 to 2.76)
Low SDI	0.32 (0.12 to 0.52)	1.5 (1.38 to 1.62)	8.17 (3.23 to 13.2)	1.33 (1.22 to 1.45)
Andean Latin America	1.15 (0.49 to 1.93)	1.81 (1.69 to 1.94)	28 (12.28 to 47.21)	1.67 (1.55 to 1.79)
Australasia	2.01 (0.85 to 3.2)	−0.78 (−0.86 to −0.71)	46.65 (20.18 to 74.39)	−0.94 (−1.02 to −0.85)
Caribbean	1.55 (0.66 to 2.56)	1.68 (1.62 to 1.73)	37.94 (16.17 to 62.94)	1.66 (1.61 to 1.71)
Central Asia	0.96 (0.41 to 1.53)	0.1 (−0.03 to 0.22)	24.46 (10.4 to 38.7)	−0.22 (−0.32 to −0.13)
Central Europe	3.03 (1.35 to 4.85)	0.56 (0.42 to 0.69)	68.94 (30.82 to 110.6)	0.48 (0.34 to 0.61)
Central Latin America	1.26 (0.56 to 2.06)	2.11 (2.04 to 2.19)	32.79 (14.66 to 52.33)	2.32 (2.25 to 2.4)
Central Sub-Saharan Africa	0.49 (0.18 to 0.83)	2.41 (2.24 to 2.57)	12.19 (4.62 to 21.03)	2.34 (2.19 to 2.5)
East Asia	0.96 (0.4 to 1.59)	2.41 (2.34 to 2.48)	24.42 (10.09 to 40.83)	2.31 (2.21 to 2.41)
Eastern Europe	2.53 (1.09 to 4.04)	0.72 (0.6 to 0.84)	60 (25.94 to 95.33)	0.45 (0.31 to 0.58)
Eastern Sub-Saharan Africa	0.48 (0.18 to 0.8)	1.62 (1.52 to 1.72)	11.97 (4.57 to 19.8)	1.33 (1.23 to 1.43)
High-income Asia Pacific	0.83 (0.32 to 1.31)	0.32 (0.27 to 0.36)	19.32 (7.65 to 30.53)	0.13 (0.09 to 0.18)
High-income North America	1.95 (0.87 to 3.03)	−0.66 (−0.76 to −0.55)	49.52 (22.63 to 76.52)	−0.42 (−0.5 to −0.33)
North Africa and Middle East	1.31 (0.58 to 2.1)	1.7 (1.54 to 1.85)	31.83 (13.68 to 50.63)	1.46 (1.32 to 1.6)
Oceania	0.59 (0.25 to 0.96)	0.51 (0.42 to 0.6)	15.48 (6.43 to 25.08)	0.44 (0.37 to 0.52)
South Asia	0.2 (0.08 to 0.32)	2.81 (2.76 to 2.86)	5.67 (2.22 to 8.92)	2.74 (2.69 to 2.78)
Southeast Asia	0.62 (0.25 to 1)	3.04 (2.93 to 3.15)	16.64 (6.73 to 26.92)	2.84 (2.72 to 2.97)
Southern Latin America	2.52 (1.12 to 4.11)	0.9 (0.73 to 1.08)	58.65 (26.14 to 95.15)	0.95 (0.8 to 1.11)
Southern Sub-Saharan Africa	1.49 (0.63 to 2.34)	2.32 (2.06 to 2.58)	35.73 (15.05 to 55.83)	2.36 (2.1 to 2.62)
Tropical Latin America	1.42 (0.6 to 2.29)	1.87 (1.76 to 1.98)	36.04 (15.2 to 57.63)	1.89 (1.79 to 2)
Western Europe	1.77 (0.75 to 2.9)	−0.65 (−0.71 to −0.59)	39.08 (16.77 to 63.51)	−0.67 (−0.74 to −0.6)
Western Sub-Saharan Africa	0.47 (0.19 to 0.75)	2.39 (2.33 to 2.45)	10.79 (4.35 to 17.69)	2.2 (2.14 to 2.25)

**Table 2:** ASMR and ASDR attributable to HFPG in 2021, and their trends from 1990 to 2021 by SDI regions and global burden of disease locations.

Location	ASMR in 2021(per 100,000)	EAPC of ASMR, 1990 to 2021 (95%CI)	ASDR in 2021 (per 100,000)	EAPC of ASDR, 1990 to 2021(95%CI)
Global	0.98 (0.51 to 1.49)	0.32 (0.23 to 0.4)	20.31 (10.46 to 30.81)	0.31 (0.24 to 0.38)
High SDI	1.33 (0.67 to 1.99)	−0.08 (−0.18 to 0.02)	27.65 (14.2 to 41.32)	−0.08 (−0.18 to 0.01)
High-middle SDI	1.17 (0.61 to 1.8)	0.7 (0.55 to 0.85)	24.87 (12.71 to 38.46)	0.63 (0.51 to 0.75)
Middle SDI	0.77 (0.4 to 1.2)	0.84 (0.79 to 0.9)	16.92 (8.68 to 26.32)	0.83 (0.78 to 0.87)
Low-middle SDI	0.52 (0.26 to 0.79)	1.86 (1.82 to 1.9)	11.71 (5.82 to 17.78)	1.79 (1.76 to 1.82)
Low SDI	0.44 (0.22 to 0.69)	0.84 (0.73 to 0.95)	9.27 (4.47 to 14.31)	0.6 (0.49 to 0.71)
Andean Latin America	0.77 (0.38 to 1.21)	2.42 (2.26 to 2.59)	15.38 (7.52 to 24.39)	2.33 (2.19 to 2.47)
Australasia	1.18 (0.61 to 1.72)	−0.73 (−0.8 to −0.67)	24.9 (12.66 to 37.04)	−0.87 (−0.94 to −0.8)
Caribbean	1.38 (0.68 to 2.17)	0.97 (0.93 to 1.02)	28.77 (14.44 to 45.74)	1.12 (1.08 to 1.17)
Central Asia	0.54 (0.26 to 0.83)	1.96 (1.73 to 2.19)	12.06 (5.85 to 18.57)	1.57 (1.38 to 1.75)
Central Europe	2.16 (1.11 to 3.25)	1.29 (1.13 to 1.45)	44.68 (22.83 to 66.76)	1.26 (1.1 to 1.42)
Central Latin America	0.87 (0.44 to 1.34)	1.32 (1.23 to 1.41)	19.66 (10.17 to 29.88)	1.61 (1.53 to 1.69)
Central Sub-Saharan Africa	0.58 (0.28 to 0.96)	0.63 (0.44 to 0.81)	12.72 (6.1 to 20.82)	0.62 (0.44 to 0.81)
East Asia	0.92 (0.47 to 1.44)	0.36 (0.22 to 0.51)	20.56 (10.15 to 31.98)	0.36 (0.25 to 0.46)
Eastern Europe	1.06 (0.54 to 1.61)	1.49 (1.39 to 1.59)	23.09 (11.8 to 34.79)	1.18 (1.07 to 1.3)
Eastern Sub-Saharan Africa	0.54 (0.26 to 0.85)	0.75 (0.62 to 0.88)	10.34 (4.98 to 16.3)	0.41 (0.26 to 0.56)
High-income Asia Pacific	1.29 (0.65 to 1.93)	−0.46 (−0.56 to −0.37)	25.57 (12.97 to 38.3)	−0.64 (−0.75 to −0.53)
High-income North America	1.41 (0.72 to 2.12)	−0.01 (−0.18 to 0.16)	31.31 (16.16 to 46.55)	0.08 (−0.06 to 0.22)
North Africa and Middle East	0.91 (0.45 to 1.41)	2.13 (1.95 to 2.31)	19.34 (9.52 to 29.77)	2 (1.84 to 2.17)
Oceania	0.59 (0.3 to 0.94)	0.29 (0.21 to 0.37)	13 (6.65 to 20.41)	0.3 (0.25 to 0.35)
South Asia	0.39 (0.19 to 0.59)	1.18 (1.07 to 1.28)	9.07 (4.57 to 13.65)	1.1 (0.99 to 1.21)
Southeast Asia	0.92 (0.46 to 1.49)	1.88 (1.8 to 1.96)	19.16 (9.69 to 30.7)	1.81 (1.75 to 1.87)
Southern Latin America	1.56 (0.79 to 2.41)	1.35 (1.15 to 1.56)	31.83 (16.18 to 48.88)	1.38 (1.19 to 1.58)
Southern Sub-Saharan Africa	0.86 (0.4 to 1.33)	2.26 (1.96 to 2.56)	17.44 (8.34 to 26.87)	2.61 (2.28 to 2.93)
Tropical Latin America	0.95 (0.49 to 1.4)	1.66 (1.56 to 1.75)	21.25 (10.86 to 31.78)	1.84 (1.74 to 1.94)
Western Europe	1.15 (0.57 to 1.77)	−0.28 (−0.38 to −0.18)	22.7 (11.26 to 34.71)	−0.29 (−0.4 to −0.18)
Western Sub-Saharan Africa	0.4 (0.19 to 0.64)	2.1 (2 to 2.21)	7.92 (3.67 to 12.51)	2 (1.91 to 2.1)

In 2021, the global ASMR for CRC attributable to HFPG was 0.98/100,000 (95% UI:0.51–1.49), and the ASDR was 20.31/100,000 (95% UI:10.46–30.81). The EAPC values for ASMR was 0.32 (95% CI:0.23–0.4) and for ASDR was 0.31 (95% CI:0.24–0.38), reflecting a slow but steady rise in the global burden. A clear divergence in EAPC trends emerged across SDI categories. High-SDI countries recorded higher ASMR (1.33/100,000; 95% UI:0.67–1.99) and ASDR (27.65/100,000; 95% UI:14.20–41.32) compared to low-SDI countries (ASMR: 0.44/100,000; 95% UI:0.22–0.69; ASDR: 9.27/100,000; 95% UI:4.47–14.31). However, but the EAPC values showed opposing directions: high-SDI countries exhibited a slight decline or stabilization, with EAPCs of −0.08 (95% CI: −0.18-0.02) for ASMR and −0.08 (95% CI: −0.18 to 0.01) for ASDR. However, the low SDI countries demonstrated a significant increase, with EAPCs of 0.84 (95% UI:0.73–0.95) for ASMR and 0.60 (95% UI:0.49–0.71) for ASDR. Regionally, Central Europe was again the highest ASMR (2.16/100,000; 95% UI:1.11–3.25) and ASDR (44.68/100,000 95% UI:22.83–66.76), whereas Western Sub-Saharan Africa was the lowest (ASMR: 0.40/100,000; 95% UI:0.19–0.64; ASDR: 7.92/100,000; 95% UI:3.67–12.51). The most rapid EAPC growth was observed in Southern Sub-Saharan Africa, with value of 2.26 (95% CI: 1.96–2.56) for ASMR and 2.61 (95% CI: 2.28–2.93) for ASDR, suggesting that its burden of hyperglycemia-associated cancers is a serious challenge (Figure 6B; Table 2).

### 3.7 Predictive analysis: forecasting the trend of DALYs and deaths associated with HBMI with HFPG from 1990 to 2035

**Figure 7:**
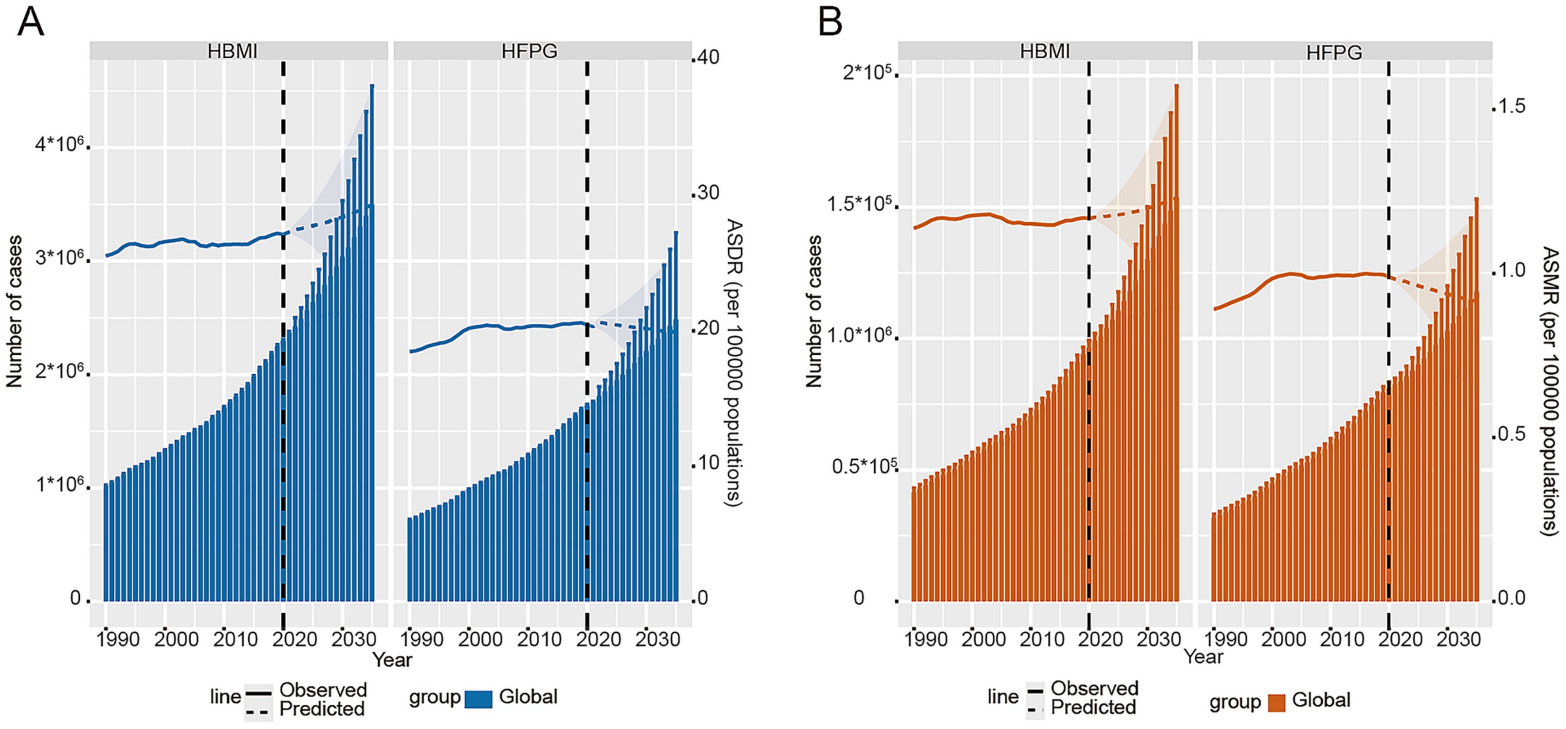
Predictive analysis. **(A)** Global total number of CRC DALYs and ASDR due to HBMI and HFPG and its projected trend by 2035. **(B)** Global BAPC projected trends in the burden of CRC deaths due to HBMI and HFPG. The vertical axis of the left panel shows total deaths; the vertical axis of the right panel shows age-standardized mortality rates; shaded areas indicate 95% uncertainty intervals.

Figure 7A show the time-series trends of the total number of CRC DALYs due to HBMI with HFPG (left) versus the age-standardized rate (ASDR, right) globally, including the observed data from 1990 to 2021 versus the projected results for the period 2022–2035. In terms of absolute numbers, the total number of DALYs due to HBMI is projected to rise from about 2364604.57 (95% UI:2349417.75–2379791.39) cases in 2021 to about 3497205.13 (95% UI:2449914.87–4544495.4) cases by 2035, an increase of about 47.90%. In contrast, the total number of DALYs due to HFPG will increase from 1750989.02 (95% UI:1738422.62–1763555.42) cases to 2485400.3 (95% UI:1719757.63–3251042.97) cases, an increase of approximately 41.94% (Figure 7A; Supplementary Table S6).

Figure 7B shows the projected trends in the number of CRC deaths and ASMR due to HBMI and HFPG based on the BAPC model globally from 2022 to 2035. In terms of total deaths due to HBMI, the estimate of 99,249 (95% UI: 96,501–101,996) in 2021 is expected to rise to approximately 153634.22 (95% UI: 111130.27 to 196138.18) in 2035, with a stable trend in average annual growth. Overall, the total number of HBMI-associated CRC deaths globally showed a steady increase. In terms of total deaths due to HFPG, the number of HFPG-associated CRC deaths were approximately 31,877 (95% UI: 30,504–33,251) in 1990, and it has risen to approximately 82,502 (95% UI: 80,133–84,872) by 2021. According to model projections, this number is expected to increase to approximately 117853.71 (95% UI: 82587.16 to 153120.27) by 2035, showing a continuous increase (Figure 7B; Supplementary Table S6). The projected increases are accompanied by wide 95% uncertainty intervals, reflecting the inherent uncertainty in long-term forecasting, particularly for absolute numbers driven by demographic changes.

## 4 Discussion

In this study, we systematically assessed the global burden and trends of CRC due to HBMI and HFPG from 1990 to 2021 using data from the GBD 2021 study and combined with predictive modeling to extrapolate the trend of burden change by 2035. Through multidimensional decomposition and stratified analysis of DALYs and mortality, we reveal a complex risk landscape shaped by demographic factors, epidemiologic shifts, and geographic heterogeneity in conjunction with the level of social development.

The main findings of the study are as follows: First, the burden of CRC due to HBMI and HFPG has continued to increase over the past three decades, primarily driven by population growth and aging. These factors remain the primary drivers of increases in both DALYs and death burden, which is consistent with established global burden of disease studies (32, 33). Second, the drivers of burden changes varied across SDI regions. Although epidemiological changes contributed less overall than demographic factors, their relative contribution to DALYs and death burden was significantly higher in middle-SDI regions. Nearly one-fifth of the increase in HFPG-associated DALYs in low- and middle-SDI region was due to epidemiological changes, reflecting rapidly increases in obesity and diabetes and the rapid westernization of lifestyles and diets (34–36), which have exacerbated the burden. Therefore, low- and middle-SDI countries should urgently implement community-based nutrition and physical activity promotion programs to counter Westernized lifestyles. In contrast, in high-SDI countries, epidemiological change—such as improved early screening and risk factor control—had a negative impact on DALYs and deaths, indicating that effective management of metabolic risks (e.g., weight management, glucose control) and widespread CRC screening have offset some of the burden from aging populations (10, 37). This trend also supports the observations of existing studies on the effectiveness of early cancer intervention in high-income countries (5, 38). Third, although the concentration index indicates that the burden remains concentrated in high-SDI countries, health inequality has decreased. The burden of CRC associated with HBMI and HFPG is trending downward in the concentration of high SDI countries. Particularly for DALYs, the BMI-related concentration index declined from 0.5670 in 1990 to 0.4575 in 2021. This change may partly benefit from the rising burden of disease in middle-SDI countries and the improved effectiveness of burden management in high-SDI countries (39). However, the limited decline in the concentration index for HFPG-related burden suggests that the contribution of hyperglycemia to the CRC burden is still concentrated in more developed countries and that the efficacy of their metabolic disease management systems has not yet produced significant spillover effects (40). In addition, in the spatial distribution of DALY and mortality, although the gap between countries with different SDIs has narrowed, it has grown faster in the lowest SDI countries, and overall health inequality has shown a deepening of the disparity structure in convergence (41, 42). This suggests that public health strategies should not only focus on changes in the overall mean but also on policy intervention windows in countries with rising risks (43, 44).

Country-level data on DALYs and the burden of death in 2021 show that the burden is generally high in Europe, the United States, and Pacific Island countries, with some Central and Eastern European nations such as Hungary, Slovakia, and Bulgaria also ranking highly. High-burden countries typically exhibit a combination of high obesity rates, low levels of physical activity, and Western diets; Pacific Island countries having a particularly high prevalence of obesity (45, 46). In contrast, countries such as India, Bangladesh, and Malawi report lower rates of DALYs and mortality. While this may be partly attributable to lower BMI versus FPG levels, it is also important to consider limitations within the data system—such as low coverage of cancer registries and misreporting of cause of death (47–49). China and Taiwan share similar cultural backgrounds and population genetic characteristics; however, differences in healthcare systems, distribution of medical resources, and availability of screening infrastructure may profoundly impact the actual manifestation of metabolic cancer burdens—resulting in variations in DALY rates (50, 51). The EAPC results reveal that BMI- and HFPG-related DALYs along with mortality rates are rapidly increasing in low- and middle-income countries such as Vietnam, Lesotho, and Egypt. These nations are undergoing rapid urbanization and nutritional transition where traditional high-fiber diets are gradually being replaced by high-fat/high-sugar diets while screening processes for metabolic disease management systems remain underdeveloped (52). This trend aligns with previous epidemiological findings indicating rapid growth in metabolic diseases across certain some South Asian countries (53). Comparatively, high-income countries like Austria, the Czech Republic, and Singapore have experienced significant declines in burden, reflecting the long-term effectiveness of their cancer screening, health promotion programs, and early diabetes interventions (54). This also suggests that the rising burden of metabolism-related cancers is not irreversible through systematic strategic interventions, even in the context of relatively uncontrollable aging (55).

In addition, The burden is significantly increases with age, peaking in the 65–69 age group. For both DALYs and mortality, the burdens of HBMI and HFPG peak in people aged 60 years or older, reflecting the time-lag effect of the impact of metabolic chronic diseases, with risk exposure occurring in midlife and cancer onset and death being more common in old age (56, 57). Therefore, policy interventions should not only focus on the older adults but also extend to the midlife window of metabolic control to reduce the future cancer burden through early screening and lifestyle modifications (58, 59). Gender analysis indicates that men bear a significantly higher burden of CRC associated with HBMI and HFPG compared to women, which is linked to their higher obesity rates, smoking, alcohol consumption, and poorer adherence to health management practices (60). This gender difference is particularly significant in high-SDI countries, suggesting that men may have lower utilization of health services despite broad coverage of current screening strategies and highlight the need for gender-sensitive health interventions (61, 62). Developing male-friendly health communication and outreach strategies could help increase participation in preventive services.

The BAPC model predicts that by 2035, the total number of DALYs will increase by approximately 47.90% due to HBMI and by 41.94% due to HFPG. However, age-standardized rates are expected to remain stable or even slightly decline, indicating that the increase in the total burden is mainly due to population growth and aging. This trend implies that the absolute disease burden will increase and healthcare resource needs will expand accordingly, even if ASDR and ASMR remain stable amid the dual challenges of population aging and metabolic disease epidemics (63, 64). Meanwhile, the slight downward trend in HFPG-associated ASMR may suggest that current diabetes control measures have begun to bear fruit in some countries, and that future promotion of intervention strategies (e.g., HbA1c screening, universal glycemic control initiatives) will have an indirect inhibitory effect on CRC burden (65). Therefore, scaling up structured glycemic control programs and integrating them with non-communicable disease prevention platforms could further reduce future CRC burden.

This study has several significant strengths. First, based on the comprehensive and systematically integrated data provided by the GBD 2021 database, we were able to systematically disaggregate the burden of CRC associated with HBMI and HFPG at the global, SDI, and national levels, bridging the gap of previous studies that focused only on the general trend but neglected the differences in drivers. Second, this study adopted a multidimensional inequality analysis to systematically reveal the spatial distribution pattern and evolutionary trend of the health burden, providing empirical evidence for resource allocation at the global and regional levels. Furthermore, we combined the BAPC model to predict the burden by 2035, which enhances the foresight and policy value of the study and helps governments and international organizations to formulate medium- and long-term prevention and control strategies. However, there are several limitations to this study. First, the GBD estimation itself is dependent on the quality of data from each country, and some low-income countries have problems such as poor cancer registries and unclear categorization of causes of death, which may lead to an underestimation of the burden of disease. Second, although this study conducted an independent assessment of HBMI and HFPG, and the GBD Comparative Risk Assessment Framework also estimates the independent effects of the two to reduce double-counting, HBMI and HFPG often coexist and may have interactions that cannot be fully quantified in this study. Therefore, there may still be residual overlapping attributes in their estimated burdens, and these estimates should be understood as the potential burdens avoidable by addressing each risk factor individually. Third, residual confounding from unmeasured lifestyle factors (diet, physical activity) may influence associations. Fourth, the BAPC model predictions were limited by past trends and assumptions, and the predictions remain uncertain in the context of rapidly changing policies and healthcare, especially if screening coverage or level of health interventions in each country changes significantly in the next 10 years.

Sustainable Development Goal 3.4 (SDG3.4) is an important international target aimed at reducing premature deaths caused by non-communicable diseases (NCDs). However, progress in this regard has been relatively slow globally, especially in low-and middle-SDI countries. This study warns about the progress made toward SDG3.4. The global burden of CRC is constantly increasing due to HBMI and HFPG, which are largely driven by demographic transitions and lifestyle changes. Strengthening metabolic risk management and expanding equitable CRC screening programs—especially in low-and middle-SDI regions - is crucial for reducing future disease burdens. To promote the achievement of SDG3.4, it is necessary to strengthen the promotion and education of healthy lifestyles globally, standardize the management of diabetes and obesity, and actively promote the popularization of physical activities among the general population. Secondly, international aid and cooperation projects should prioritize supporting low- and middle-SDI countries in establishing and improving early screening systems for CRC and incorporating BMI and blood glucose screening into primary CRC programs, particularly to increase the participation rate of men over 60 in screening. For high-burden regions, targeted prevention strategies should be developed based on local risk factors (such as diet, genetic factors, and the medical system). Additionally, the construction of elderly medical service systems should be accelerated to meet the growing demand for cancer patient care due to the increasing population aging. Only in this way can the trend of rising non-communicable disease burdens caused by population aging and risk factors be reversed, and the SDG 3.4 plan can be achieved.

## Data Availability

The datasets presented in this study can be found in online repositories. The names of the repository/repositories and accession number(s) can be found in the article/Supplementary material.
