# Supplementary material for: Decomposition and forecasting of colorectal cancer burden attributable to high body mass index and high fasting plasma glucose, 1990–2021: A GBD 2021 study
Source: Front Nutr. 2025 Dec 17;12:1652676. doi: 10.3389/fnut.2025.1652676 (PMC12753452; doi:10.3389/fnut.2025.1652676)
Supplement: Supplementary file 1 [file Data_Sheet_1.docx]

**Figure S1: Flowchart of the analytical framework**


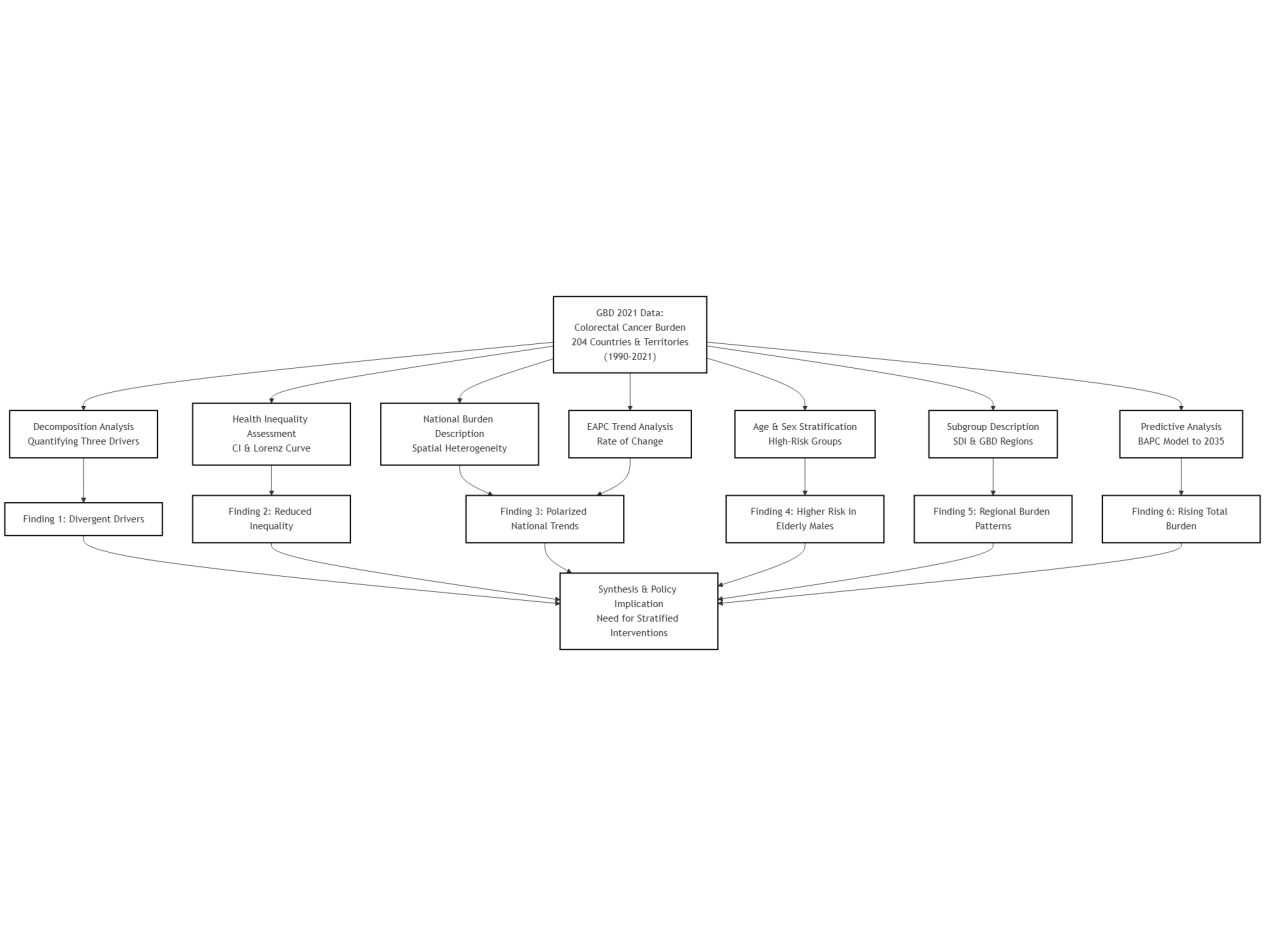


**Table S1.** Concentration Index of Disease Burden Attributable to HBMI and HFPG for Colorectal Cancer, by Year, Age Group, and Risk Factor.

**Table S2.** Slope Index and concentration index of Inequality in Disease Burden Attributable to HBMI and HFPG for Colorectal Cancer.

**Table S3.** Analysis of differences in the burden of DALYs, deaths and EAPC trend at country level.

**Table S4.** Analysis of the burden of DALYs and deaths by age group.

**Table S5.** Analysis of the burden of colorectal cancer by gender.

**Table S6.** The trend of DALYs and deaths from 1990 to 2035.
